# Tumor Burden and Intraosseous Metabolic Activity as Predictors of Bone Marrow Failure during Radioisotope Therapy in Metastasized Prostate Cancer Patients

**DOI:** 10.1155/2017/3905216

**Published:** 2017-12-25

**Authors:** Francesco Fiz, Samine Sahbai, Cristina Campi, Matthias Weissinger, Helmut Dittmann, Cecilia Marini, Michele Piana, Gianmario Sambuceti, Christian la Fougère

**Affiliations:** ^1^Nuclear Medicine Unit, Department of Radiology, University of Tübingen, Tübingen, Germany; ^2^Department of Internal Medicine, University of Genoa, Genova, Italy; ^3^Nuclear Medicine Unit, “Henri Mondor” University Hospital, Paris, France; ^4^National Council of Research-SPIN, Section of Genoa, Genova, Italy; ^5^National Council of Research-IBFM, Section of Genoa, Genova, Italy; ^6^Department of Mathematics, University of Genoa, Genova, Italy; ^7^Nuclear Medicine Unit, Department of Health Sciences, University of Genoa, Genova, Italy

## Abstract

**Rationale:**

Radium-223-Dichloride (Ra-223) is an alpha-emitter, used to treat bone metastases. Patients with high metastatic burden and/or with increased trabecular bone uptake could present a higher incidence of hematologic toxicity. We hypothesized that these two factors are predictors of bone marrow failure.

**Material and Methods:**

A computer algorithm discriminated between trabecular bone (*B*_Vol_) and tumor metastases (*M*_Vol_) within pretherapeutic whole-body skeletal SPECT/CT (*N* = 47). The program calculated the metastatic invasion percent (INV%) as the *M*_Vol_/(*M*_Vol_ + *B*_Vol_) ratio and extracted the *B*_Vol_ mean counts. *B*_Vol_ counts were correlated to % drop of hemoglobin (Hb), leukocytes (WBC), and platelets (PLT) after 3/6 Ra-223 cycles. Patient-specific and computational-derived parameters were tested as predictors of hematologic toxicity with MANOVA.

**Results:**

*B*_Vol_ counts correlated with drop of Hb (*R* = 0,65, *p* < 0.01) and PLT (*R* = 0,45, *p* < 0.01). Appendicular *B*_Vol_ counts showed a better correlation (*p* < 0.05, *p* < 0.01, and *p* < 0.001 for Hb, WBC, and PLT, resp.). INV% directly correlated with *B*_Vol_ counts (*R* = 0.68, *p* < 0.001). At MANOVA, grade III/IV toxicity was predicted by INV% (*p* < 0.01), by long-bone invasion (*p* < 0.005), and by *B*_Vol_ counts (*p* < 0.05).

**Conclusions:**

In patients with significant bone tumor burden, degree of bone invasion and trabecular bone uptake are predictors of subsequent bone marrow failure.

## 1. Introduction

Patients affected by castration-resistant prostate cancer (CRPC) present an extremely high incidence of skeletal metastases [1]. In recent years, numerous novel treatment modalities have been developed to treat metastasized CRPC [2–5]. Among these, radionuclide therapy with alpha-emitting Ra-223-Dichloride (Ra-223) has been recently approved [6]. This isotope allows obtaining a significant survival improvement, alongside with satisfying pain palliation and reduction of skeletal-related events [7–11]. Moreover, Ra-223 therapy, owing to its short emission radius [12], is characterized by a relatively low incidence of hematologic toxicity, when compared with *β*-emitting radioisotopes [13, 14]. In fact, Ra-223-related bone marrow failure presents an incidence of 3-4% for grade III and 1% for grade IV reactions [10].

However, these data are derived from a heterogeneous CRPC population, whose patients presented very diverse tumor burden, ranging from 2 to >20 metastases. Actually, more than half of the enrolled population had less than 20 skeletal localizations [10]. Recent studies have however suggested that the incidence of bone marrow failure could be more common in patients presenting a higher number of skeletal metastases [6, 15, 16].

Two main factors could influence the onset of bone marrow toxicity during the Ra-223 therapy: the tumor burden per se and the metabolic intensity of nontarget trabecular bone.

In fact, under normal conditions, active bone marrow takes up about one-third of the available volume within the intrabone space; this “red” bone marrow is located within the vertebrae, the sternum, and the flat bones [17]. If this space is invaded by metastases, hematopoietic stem cells can home and seed in more distal hematopoietic niches in the appendicular skeleton. As the disease inevitably progresses, bone metastases are increasingly observed in the appendicular skeleton as well. In this scenario, the space for healthy bone marrow becomes also restricted and necessary compensatory bone marrow expansion (e.g., because of Ra-223 induced damage) is hampered.

On the other hand, radiotracer uptake within the trabecular skeleton, where the hematopoietic bone marrow is located, can present significant interpatient variations [18, 19]. As Ra-223 uptake tends to mirror the one of the common bone-seeking tracers [8, 20], a higher metabolism in the pretherapeutic bone scintigraphy could entail a greater Ra-223 uptake as well as a longer residence time within the trabecular bone, possibly resulting in an overall higher dose to the bone marrow.

The first point, that is, correlation between tumor burden and bone marrow failure, was tested by studies employing an automated thresholding in bone scans [21] or in PET [22]. Though interesting, the PET-threshold approach is scanner-specific, requiring center-specific validation as well as periodic recalibrations, and is therefore not readily exportable; the planar analysis is on the other hand subject to summation artifacts [23].

With regard to the second point, the correlation between bone tracer uptake intensity and hematologic toxicity remains to this day substantially unexplored.

In this paper, we applied a novel computational tool to hybrid SPECT/CT imaging, to measure the overall tumor volume and the radioactivity concentration in the bone marrow. This method uses a segmentation algorithm to identify the trabecular bone and the metastases in the CT images, thus avoiding the problems related to PET/scintigraphy thresholding. Then it can extract additional data from the coregistered metabolic images, in order to estimate the tracer concentration in these two volumes. The goal of the present investigation is to test the hypothesis that an enhanced intrabone mineral metabolism and an increased tumor burden can predict a higher risk for bone marrow toxicity. Moreover, the specific role of the bone marrow located within the appendicular skeleton, which might be key in patients with extensive axial bones metastasization, is also explored.

## 2. Materials and Methods

### 2.1. Patients' Population and Procedures

Forty-seven adult patients (mean age 69.5 ± 7, age range 55.5–80.8), consecutively admitted to our unit for pretherapeutic evaluation of skeletal metastases from CRPC were retrospectively analyzed. Inclusion criteria comprised histologically confirmed prostate neoplasia, evidence of prostate specific antigen (PSA) increase in course of maximal androgen blockade, and presence of clinically symptomatic and radiological confirmed skeletal metastases. Exclusion criteria were confirmed or suspected visceral metastases, presence of grade 2 hematologic toxicity at baseline, according to CTCAE criteria, version 4.0 [24], end-stage renal disease, imminent danger of pathologic fracture or unwillingness/incapacity to sign an informed consent. Any previous therapy or combination of treatments was admitted. Similarly, any entity of metastatic burden was conceded and no selection on the basis of number of metabolically active areas was performed.

Each patient underwent a baseline Tc-99m-dicarboxypropane-diphosphonate (DPD) whole-body bone SPECT/CT scan, followed by six Ra-223-Dichloride injections (Xofigo®, Bayer Pharma AG, Berlin, Germany, 50 KBq per Kg of body weight), each spaced one month apart. Hemoglobin, leukocytes (WBC), and platelets (PLT) were monitored on a weekly basis. Percent variation of these parameters was calculated after three and six Ra-223-Dichloride cycles.

All patients gave written informed consent for the retrospective analysis of the pseudonymized clinical SPECT/CT data. The investigations were conducted in accordance with the Helsinki Declaration and with national regulations, after approval by the ethics committee of the University of Tübingen.

### 2.2. Scan Protocol

Patients were scanned on a hybrid SPECT/CT device (Discovery 670 Pro, GE Healthcare, Chicago, US), three hours after injection of 8–10 MBq/Kg of Tc-99M-DPD (CIS Bio, Berlin, Germany). To minimize artifacts caused by the presence of radioactive urine in the excretory system, patients were asked to drink at least 1000 ml of water during the uptake time and to void immediately before the scan.

The acquisition comprised a whole-body planar scan, followed by a whole-body SPECT/CT, from vertex up to the distal femoral epiphyses, obtained by reconstructing and fusing three sequential fields of view (Xeleris 3, GE Healthcare, Chicago, USA). SPECT acquisition was carried out with the two camera heads in H-Mode; parameters for each field of view were as follows: energy window 140.5 ± 10%, angular step 6°, time per step 15′′. The transaxial field of view and pixel size of the reconstructed SPECT images were 54 cm and 5 × 5 mm, respectively, with a matrix size of 128 × 128. SPECT raw data were reconstructed using OSEM iterative protocol (2 iterations, 10 subsets).

The 16-detector row, helical CT scanner used a gantry rotation speed of 0.8 s and a table speed of 20 mm per rotation, with a 120 kV voltage and 10–80 mA current. A dose modulation system (OptiDose, GE Healthcare, Chicago, US) was applied to minimize total exposure according to the patient's size. No contrast medium was injected.

### 2.3. Image Analysis

Segmentation of bone volumes on the CT images was based on the previously validated method [17, 25]. The computational software analysis tool was progressively developed and adapted by the joint effort of the Department of Mathematics and the Department of Health Sciences of the University of Genoa [17, 25–27]; it was later improved thanks to the collaboration with the Nuclear Medicine Unit of the University of Tübingen. The algorithm is based on the principle of segmentation analysis with adaptive threshold: it segments the osseous tissue not by aprioristically defining a Hounsfield value for bone, but by rather identifying the bone border, on each separate slice, by recognizing the sharp variation of attenuation value between soft and bone tissue.

**Figure 1:**
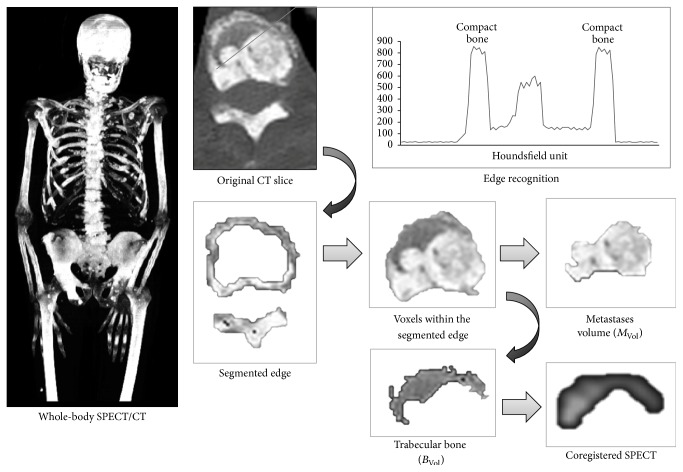
Segmentation analysis of SPECT/CT images. The program detects the compact bone border, on a slice-by-slice basis, by identifying the abrupt change of Hounsfield value (segmented edge). The mean density value of this volume is used to tell apart the trabecular bone (*B*_Vol_) from the metastases volume (*M*_Vol_) on the voxels lying inside the detected surface. The program then generates two masks, corresponding to *B*_Vol_ and to *T*_Vol_, which are exported to the coregistered SPECT images, from which the functional information is extracted.

Briefly, the algorithm identifies the skeleton on CT images by recognizing the sharp attenuation contrast between soft tissues and outer cortical bone. In normal subjects, two main components are identified: the outer compact bone and the intraosseous cancellous tissue, which can be recognized by the program in virtue of the attenuation shift between cortical and trabecular bone (Figure 1). In patients with bone metastases, a third volume can be identified, corresponding to the bone metastases. This volume can also be segmented, using the sharp contrast with the normal trabecular bone.

The bone recognition algorithm functions in two steps: in the first step the program identifies the outer margins of compact bone; from this point, it samples a two-pixel ring of skeletal tissue. Thereafter, all skeletal voxels lying internally to the compact bone volume and having attenuation coefficient equal or above the average compact bone density are considered as tumor metastases, while the remaining ones are labeled as normal trabecular bone (Figure 1). At the end of the first step, the program separates the three bone subcomponents, the compact bone, the trabecular bone (*B*_Vol_), and the bone metastases (*M*_Vol_), and calculates their volumes. Then, it proceeds to calculate the entity of metastatic invasion (INV%), as the *M*_Vol_/(*M*_Vol_ + *B*_Vol_) ratio.

In the second step, the algorithm creates two masks, corresponding to *B*_Vol_ and to *M*_Vol_ and exports them to the coregistered SPECT images. Then, it measures the radioactivity concentration, expressed as mean counts, within these two volumes.

The whole analysis is conducted first on the whole-body skeleton. Then, separate analyses are run on the bones of the axial skeleton (vertebrae and sternum) and for the long bones in the appendicular skeleton (humeral and femoral shafts). The costae are not analyzed, so as to prevent artifacts derived from the respiratory motion.

In order to detect patients presenting massive appendicular invasion (AI), as a sign of advanced disease, patients were stratified in two groups on the basis of the median value of appendicular INV%: those in the upper half were considered to be AI-positive patients.

### 2.4. Statistical Analysis

The* t*-test for unpaired data was used to compare values between patients' subgroups. Correlation between indexes was assessed with bivariate analysis, using Pearson's *R* index.

Differences in the events' occurrence between groups were tested using *X*^2^ or Fisher's exact test, as appropriate. Multivariate analysis of variance (MANOVA) was used to test the influence of *B*_Vol_ and *M*_Vol_ counts, INV%, body weight (as it reflects the administered activity), age, PSA values, “superscan,” and AI on the occurrence of grade III/IV hematologic toxicity. A *p* value of <0.05 was considered significant. The SPSS statistical program (SPSS®, v. 21.0, IBM, Armonk NY, USA) was employed.

## 3. Results

### 3.1. Patients' Characteristics

32 out of 47 patients presented high-risk disease [28] at diagnosis (68%). Mean disease duration was 7.5 ± 5.8 years. On average, patients had known skeletal metastases since 36 ± 26 months; twelve patients (26%) had a history of skeletal-related events. Mean time since hormone resistance onset was 30 ± 23 months and mean PSA at the time of the bone scan was 788 ± 1843 ng/ml. None of the patients was therapy-naïve at the time of therapy; 34 (72%) had a history of previous docetaxel therapy and 15 of them (32%) had a history of skeletal radiation therapy. All patients were on antihormone therapy at the time of Ra-223-therapy. All patients exhibited extensive metastasization: mean number of localizations, as observable on SPECT-MIP, was 76 ± 32 (range 34–135). Patients' characteristics are detailed in Table 1.

### 3.2. Degree of Trabecular Bone Invasion and Uptake Distribution

Average INV% was 28 ± 20% (range 4–71%). This figure did not exhibit significant variation between the axial (36 ± 28%, range 4–78%) and the appendicular sites (31 ± 21%, range 1–69%).

**Figure 2:**
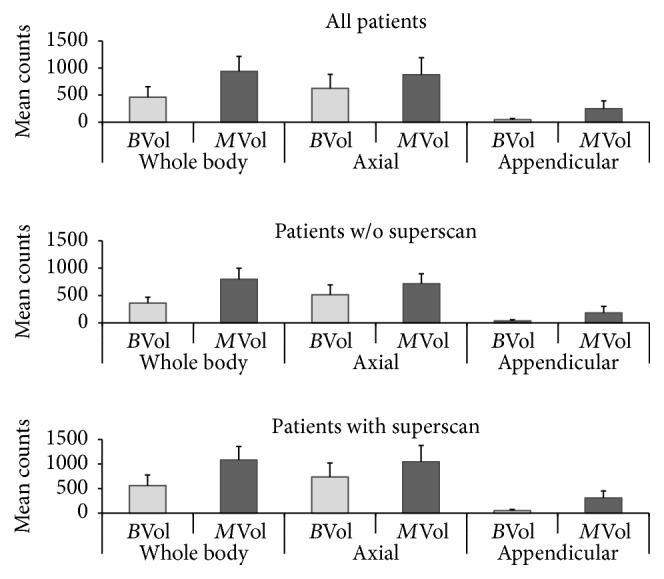
Mean counts in *B*_Vol_/*M*_Vol_ in whole-body, axial, and appendicular skeleton.

Mean counts were higher in *M*_Vol_ than in *B*_Vol_ and tended to be markedly higher in the axial than in the appendicular skeleton (Figure 2). 28 patients (59%) had a “superscan” appearance (completely of nearly absent visualization of renal parenchyma in the planar bone scan).

**Figure 3:**
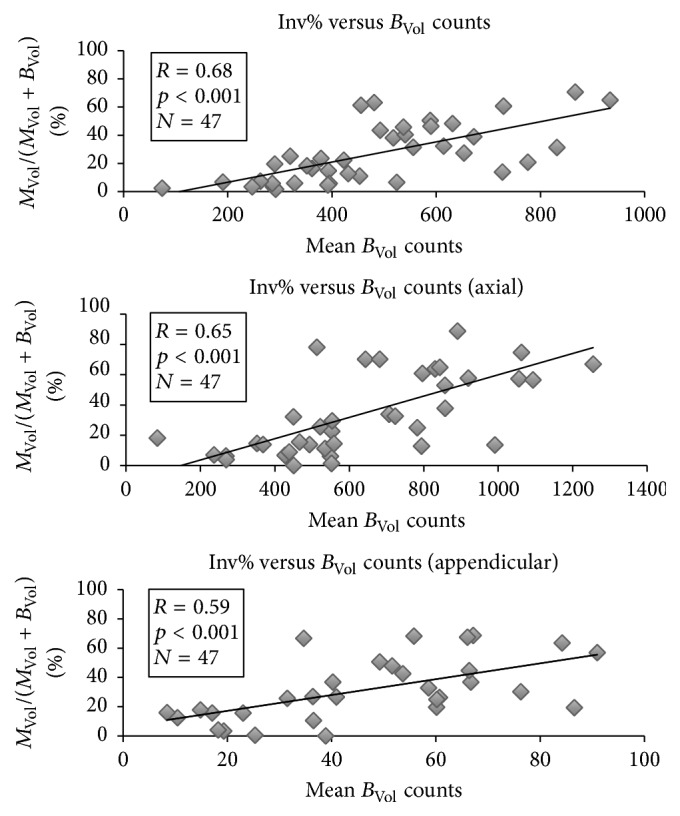
Correlation between percent metastatic invasion and mean uptake of unaffected trabecular bone. The entity of metastatic invasion positively and tightly correlates with bone uptake within trabecular bone. Said uptake can present significant variations among patients.

A direct correlation was noted between INV% and *B*_Vol_ counts (*R* = 0.68, *p* < 0.001); this correlation was also observable when considering the axial and the appendicular skeleton separately (*R* = 0.65 and *p* < 0.001; *R* = 0.59 and *p* < 0.01, resp.) as shown in Figure 3.

### 3.3. Therapy Adherence and High-Grade Bone Marrow Toxicity

All patients underwent at least one Ra-223-injection, 45 (96%) underwent the first three cycles, and 32 (75%) completed the entire six cycles of therapy. Reasons for Ra-223 discontinuation included skeletal progression at interim analysis by means of bone scan and CT (*n* = 5), spread to visceral organs (*n* = 4), occurrence of grade III or higher hematologic toxicity not amenable to control (*n* = 4), serious infection requiring hospitalization (*n* = 1), or withdrawal of consent after therapy initiation (*n* = 1). For these reasons, hematologic data after three cycles are available for all patients, while blood counts after the complete six-cycle therapy are present for 32 patients (75%) only.

After three cycles, four patients had grade III Hb toxicity; two of them discontinued the treatment while the other two continued under supportive therapy: after six cycles, one of them recovered while the other still met the grade III Hb toxicity criteria. Three further patients had developed grade III Hb toxicity after six cycles.

Grade III leukopenia/neutropenia was observed after three cycles in one patient, who subsequently recovered. One more patient developed a grade IV leukopenia/neutropenia after three Ra-223 injections.

In total, nine patients (19%) showed an at least grade III bone marrow dysfunction at some point in the course of Ra-223 treatment.

No differences in the occurrence of toxicity or in the entity of blood parameter drop were observed when categorizing the patients on the basis of previous docetaxel/radiotherapy.

### 3.4. Therapy-Associated Changes in Blood Counts

**Figure 4:**
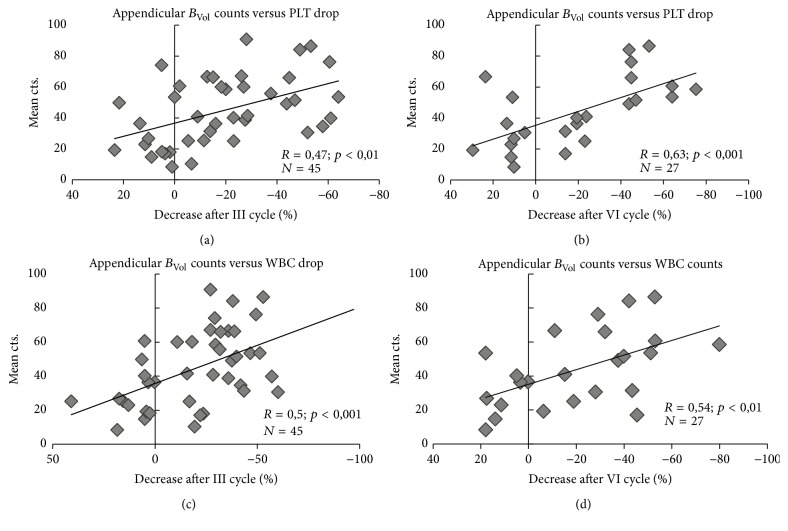
Correlation between appendicular *B*_Vol_ mean counts and blood values drop, after three (a, c) and six (b, d) cycles of Ra-223-Dichloride. PLT = platelets; WBC = leukocytes.

After three Ra-223 cycles, at least one parameter among Hb, WBC, and PLT had dropped by 10–50% in 39 patients (87%); a drop of at least 50% in any parameter was instead observed in 14 patients (30%). Correlation between whole-body *B*_Vol_ counts and drop of the above-cited parameter was direct (Hb: *R* = 0.33 and *p* < 0.05; WBC: *R* = 0.42 and *p* < 0.01 and PLT: *R* = 0.49 and *p* < 0.001). At site-based analysis, percent drop correlated with appendicular *B*_Vol_ mean counts (Hb: *p* < 0.05; WBC: *p* < 0.001 and PLT: *p* < 0.01, Figure 4). Conversely, correlation with axial *B*_Vol_ mean counts was looser and limited to WBC (*p* < 0.05).

At the end of the six cycles, 26/32 patients (81%) presented at least one parameter inferior by 10–50% with respect to baseline; 11/32 (34%) showed a ≥50% drop. Correlation between whole-body *B*_Vol_ mean counts was particularly evident when considering variation of Hb and PLT (*R* = 0.65 and *p* = 0.001 for Hb; *R* = 0.45 and *p* < 0.05 for PLT). Similar to the interim evaluation, axial *B*_Vol_ mean counts did not significantly correlate with blood element variation; conversely, appendicular mean counts showed a robust association with the end-of-therapy Hb, WBC, and PLT (*p* < 0.05, <0.01, and <0.001, resp., Figure 4). See Table 2 for the complete layout.

With the exception of a sporadic association between appendicular mean counts and interim WBC (*p* < 0.05), values of *M*_Vol_ counts in any body region were not significantly correlated with hematologic variations (data not shown).

### 3.5. Role of Appendicular Invasion (AI)

**Figure 5:**
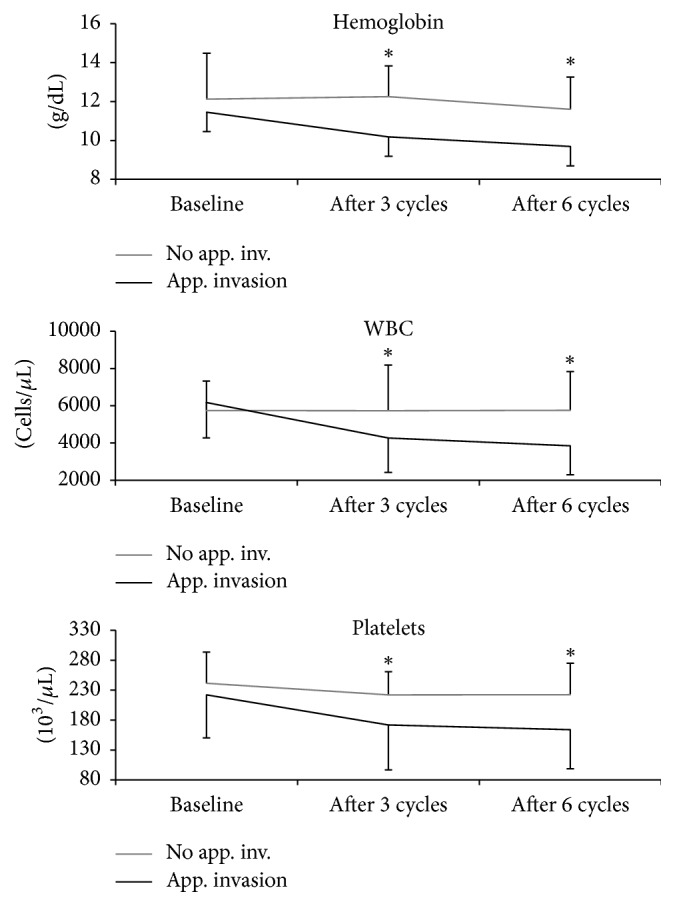
Course of blood values over time in patients with or without significant appendicular skeleton invasion. PLT = platelets; WBC = leukocytes. ^*∗*^*p* < 0.05.

A significant AI was present in 23 (49%) patients, as defined by the presence of an appendicular INV% higher than the median value (i.e., >27%). Patients with AI had a more pronounced drop of all three parameters considered (Hb, *p* < 0.05; WBC, *p* < 0.001; and PLT, *p* < 0.01) with respect to the remaining ones. Moreover, all the above-mentioned blood parameters were lower after three cycles in the AI group; this difference persisted after six cycles as well (*p* < 0.05, Figure 5). Six patients (12,7%) needed at least one erythrocytes transfusion in the course of Ra-223 therapy; all of them belonged to the AI subgroup; moreover, all patients with grade III bone marrow toxicity and the one patient with grade IV leukopenia/neutropenia belonged to this subpopulation as well (see Figure 4 for details).

### 3.6. MANOVA

INV% ratio predicted onset of grade III/IV hematologic toxicity at the whole-body (*p* < 0.05) and in the appendicular skeleton analysis (*p* < 0.01). Moreover, presence of AI predicted the onset of bone marrow toxicity (*p* < 0.005).

Similarly, mean *B*_Vol_ counts predicted grade III/IV hematologic toxicity in the whole-body as well as in the appendicular segments (*p* < 0.05) (see Table 3 for details).

## 4. Discussion

The present paper introduces a computational method, based on segmentation analysis, to estimate the skeletal tumor burden and the dose to the hematopoietic bone marrow, situated in the trabecular bone niches. Our results confirm that both metastatic invasion and delivered activity to the trabecular bone can affect the bone marrow function.

In this study, we considered a patient population that presents a relevant metastatic invasion. On average, one-third of the available intraosseous space was occupied by tumor localizations. Previous studies have shown that normal hematopoiesis roughly requires one-third of the trabecular intraosseous space [17]. In the scenario of a 30% metastatic invasion, a hematopoietic volume expansion would still be possible.

However, patients presenting a skeletal metastasization (as calculated by the INV% score) reaching the high end of the spectrum (i.e., 60–70%) could not be able to react to a cytotoxic insult. Moreover, previous chemo- or radiotherapy could have already partly impaired the bone marrow reserve. As the mechanisms of metastasization generally affect the axial skeleton first [29], the presence of a massive metastatic component within the long bone could reflect a scenario of significant skeletal invasion [30].

In order to clarify the possible mechanisms of Ra-223 toxicity, a posttherapy bone marrow dosimetry would be necessary. However, the emission spectrum of this isotope does not allow for quantification in the clinical setting, as the injected activity is low and the fraction of emitted gamma-photons is too scarce. Given the reported similarity between Ra-223 and the 99mTc-based bone-seeking tracer, we analyzed the pattern of the diagnostic tracer distribution in the cancellous bone of these subjects. The analysis showed that the trabecular bone activity could exhibit a 4-fold variation among subjects. Moreover, subjects showing a more intense uptake in the pretherapy bone SPECT/CT had a greater decline of hematologic parameters. This correlation was especially observed in the long bones, probably reflecting the above-described mechanisms, entailing a delocalization of active bone marrow from the axial skeleton to the long bones [25, 31–34], occurring in patients with considerable metastatic invasion.

As a matter of fact, the activity rate measured within the trabecular bones was strictly dependent on the entity of the metastatic invasion: the more the intraosseous spaces were occupied by tumor localizations, the higher the activity measured was in the apparently unaffected trabecular tissue. This could reflect either a tendency to instability in a skeleton whose static mechanisms are impaired by the osteoblastic reactions [18, 19, 35] or, possibly, the presence of microscopic metastatic invasion that cannot yet be demonstrated at morphological imaging [36]. Independently of the pathophysiological considerations, these data suggest that a massive presence of metastases can impair bone marrow function during radionuclide therapy in two ways: by restricting the space available for bone marrow expansion and by indirectly increasing the dose to the unaffected trabecular bone.

The hereby-presented data introduce the concept of CT-based tumor burden analysis. This method was developed so as to reduce intercenters as well as interdevices variations. Nevertheless, the concept of a correlation between tumor burden and bone marrow failure can be applied to any tumor volume measurement method [15] and to any form of radioisotope therapy (such as Sm-153).

This study presents some limitations. It is a retrospective study, enrolling a relatively low number of patients. Therefore, incidence of bone marrow toxicity that was reported here cannot be directly related to those of larger trials [10, 11]. However, previous trials considered a more diverse prostate cancer cohort, while the current study focused on the scenario of disseminated skeletal invasion. In this line, it confirms and extends on a computational perspective the results reported on planar scans in a similar cohort of patients [15].

Also, the representation of trabecular activity and blood parameter decline, as shown in Figure 3, is somewhat skewed; it must be accounted that the study analyzed a cohort of patients who had undergone different treatment scheme with a different duration before Ra-223 and whose baseline hematopoietic reserve could have been impaired by these therapies. However, the multivariate analysis confirms that trabecular tropism for bone-seeking tracers is an independent risk factor for bone marrow toxicity, at least in the setting of advanced disease.

Given the relatively low spatial resolution of SPECT, it cannot be excluded that radioactivity in osteoblastic tumor areas can at least partially contribute to the trabecular bone uptake, due to partial volume effect. This is unavoidable and represents a known limitation of the computational approach, especially when applied to SPECT/CT. However, the measured activity within metastases was not predictive of subsequent bone marrow failure, while the activity within trabecular bone is predictive. According to these considerations, the contribution of the radioactivity within the metastases is therefore less likely to have caused significant alterations on the counts within the trabecular bone. Moreover, as the segmentation method is based on CT images, it can be translated onto PET/CT data, in order to obtain a better spatial resolution.

## 5. Conclusions

Computational analysis of SPECT/CT images can estimate the degree of skeletal metastatic invasion, as well as the radioactivity concentration in the trabecular bone, where the bone marrow is located. The analysis of these parameters suggests that, in patients with a high skeletal tumor burden, the incidence of bone marrow failure in the course of radionuclide therapy could be higher than what reported previously. Further prospective studies are needed in order to confirm these data. The computational analysis could be used, in selected cases, to complement the visual evaluation and to possibly personalize the therapeutic protocols.

## Figures and Tables

**Table 1:** Patients' characteristics.

Feature	Mean	Range
Age (years)	69,5 ± 7	55–81
Weight (Kg)	72,8 ± 27,6	44–111
PSA (ng/ml)	788 ± 1843	3–9375
Gleason score	8 ± 1	5–9
Disease duration (months)	90,5 ± 69	13,7–275,8
Skeletal metastases duration (months)	36,3 ± 26	1,1–139,8
CRPC duration (months)	30 ± 23	1,9–105
Previous docetaxel	34 (72%)	-
Previous skeletal RT	12 (26%)	-
Focal lesions at SPECT MIP	76 ± 32	34–135
Mean baseline hemoglobin	12 ± 2	8,8–16,2
Mean baseline leucocytes	5876 ± 1743	3100–10940
Mean baseline platelets	220 ± 66	71–351

**Table 2:** Correlation between trabecular mean counts and parameters drop.

	*B*_Vol_ counts	AXIAL *B*_Vol_ counts	Appendicular *B*_Vol_ counts
Hemoglobin (after 3rd cycle)	−0,332^*∗*^	0,023	−0,356^*∗*^
Leucocytes (after 3rd cycle)	−0,423^*∗∗*^	−0,318^*∗*^	−0,497^*∗∗∗*^
Platelets (after 3rd cycle)	−0,479^*∗∗∗*^	−0,295	−0,473^*∗∗*^
Hemoglobin (after 6th cycle)	−0,644^*∗∗*^	−0,264	−0,412^*∗*^
Leucocytes (after 6th cycle)	−0,308	−0,108	−0,539^*∗∗*^
Platelets (after 6th cycle)	−0,451^*∗*^	−0,254	−0,628^*∗∗∗*^

^*∗*^ *p* < 0.05; ^*∗∗*^*p* < 0.01; ^*∗∗∗*^*p* < 0.001; MCs: mean counts.

**Table 3:** Results of multivariate analysis for grade III/IV hematologic toxicity.

Dependent variable	Degrees of freedom	*F* value	*p* value
Age	1	2,555	NS
Weight	1	1,978	NS
PSA level	1	3,637	NS
Superscan	1	0,33	NS
*B*_Vol_ counts	1	9,346	0,016
Axial *B*_Vol_ counts	1	0,134	NS
Appendicular *B*_Vol_ counts	1	5,318	0,045
*M*_Vol_ mean counts	1	1,374	NS
Axial *M*_Vol_ mean counts	1	0,708	NS
Appendicular *M*_Vol_ mean counts	1	2,933	NS
INV%	1	9,881	0,014
INV% (axial skeleton)	1	1,509	NS
INV% (appendicular skeleton)	1	12,366	0,008
Appendicular invasion (INV% > median)	1	16	0,004
